# Lived Experiences of New‐Onset Long Covid Pain and Its Impact on Health‐Related Quality of Life. A Scoping Review of Current Evidence

**DOI:** 10.1111/hex.70352

**Published:** 2025-07-22

**Authors:** Minimol Paulose, Nicholas Norman Adams, Kathryn R. Martin, Aileen Grant

**Affiliations:** ^1^ School of Health, Ishbel Gordon Building Robert Gordon University Aberdeen UK; ^2^ Academic Primary Care, Institute of Applied Health Science, School of Medicine, Medical Sciences and Nutrition University of Aberdeen Aberdeen UK

**Keywords:** chronic pain, long Covid, pain, post Covid, quality of life, SARS‐CoV‐2

## Abstract

**Introduction:**

Long Covid (LC) is a multisystem condition that can cause persistent symptoms such as breathlessness, fatigue, cognitive problems and pain, with major effects on individuals and healthcare systems. Globally, nearly 400 million people have been affected. New‐onset pain is among the most commonly reported symptoms and may develop into chronic pain, contributing to reduced health‐related quality of life (HRQoL) and highlighting the need for appropriate care. Given its global prevalence, exploring how people experience new‐onset LC pain and how it impacts their lives can help improve pain management and support services.

**Methods:**

A mixed‐methods scoping review was conducted following the Joanna Briggs Institute (JBI) guidance and the Preferred Reporting Items for Systematic Reviews Extension for Scoping Reviews (PRISMA‐ScR). The review mapped and synthesised evidence from eligible primary research articles (quantitative, qualitative and mixed‐methods) published in English between December 2019 and June 2024. Seven studies using cross‐sectional, case–control and observational designs (*n* = 30 to 2507 participants) were included, with data collected from Europe and Asia.

**Results:**

While qualitative data on lived experience were limited, 69.5% of LC patients reported new‐onset pain, most commonly musculoskeletal (MSK) pain (73.2%). Psychological symptoms such as post‐traumatic stress disorder (PTSD) were also reported (38%). Pain medications were widely used. Findings suggest that new‐onset LC pain affects physical, psychological and social well‐being. No studies involving children or adolescents were identified, indicating a gap in the evidence on paediatric experiences of new‐onset LC pain.

**Conclusion:**

This review highlights major gaps in the literature, especially the lack of qualitative research on how people experience new‐onset LC pain. Future research should explore these experiences in depth, with involvement from patients and the public, to inform the development of appropriate treatment and support strategies.

**Patient or Public Contribution:**

During the review process, opportunities to involve PPI were not fully explored due to limited awareness of how to support meaningful involvement in a scoping review, alongside time and resource constraints. Such involvement could have helped shape the review question, refine the search terms and interpret the findings in ways that better reflect lived experience. This is acknowledged as both a limitation and a learning point. PPI will be actively embedded in the next phases of the research.

## 1 Background

The enduring health impacts of Covid‐19 have become a major global public health concern. Post‐Covid‐19 condition, commonly referred to as long Covid (LC), is defined by the World Health Organization (WHO) as the persistence or emergence of new symptoms 3 months after a Covid‐19 infection, lasting for at least 2 months, with no alternative diagnosis [1]. It affects individuals regardless of age or the initial severity of their illness [1].

Estimates suggest that approximately 400 million people worldwide—about 6% of adults and 1% of children—have experienced LC [2]. The economic burden of LC is substantial, with an annual cost of approximately $1 trillion globally, representing about 1% of the global economy due to healthcare costs and reduced productivity [2]. Nevertheless, the vague nature of its symptoms, absence of systematic screening and lack of dependable diagnostic indicators make accurate case identification challenging, likely resulting in under‐reporting [3]. As of now, there are no validated biomarkers or approved treatment options for LC, posing difficulties for healthcare providers and increasing the frustration of those affected by LC [4, 5]. There is an urgent need for management guidelines for primary care providers, who are crucial in facilitating access to specialised care to improve the quality of life of LC patients [5].

LC has been linked to over 200 symptoms, affecting multiple organ systems [4]. These symptoms are diverse and often complex, including fatigue, breathing difficulties, cognitive dysfunctions like brain fog, and physical symptoms such as pain [2, 6, 7]. Their severity often fluctuates, or they may recur over time [8]. Pain is one of the most reported and debilitating symptoms of LC, impacting approximately 19.6% [9] to 50% of those affected by the condition [10, 11].

Research shows that a notable proportion (50.8%) of those affected by Covid‐19 experience new‐onset pain within the first month after discharge from the hospital, which is strongly associated with a lower HRQoL [12]. This pain can frequently present as musculoskeletal (MSK) pain, headache or neuropathic pain, and it might persist for several months [12, 13]—or even as long as 2 years—after the initial infection from Covid‐19 [14, 15]. This ongoing pain can greatly impact a person's ability to carry out daily activities, contribute to emotional challenges, and result in feelings of isolation [16, 17].

In this review, new‐onset pain refers to any pain that began during or after a Covid‐19 infection and was not present beforehand. While not all new‐onset pain symptoms were reported as long‐term, several studies reported that the pain continued for weeks or even months, and it can be ongoing and disruptive to daily life. This review looked at studies that matched the WHO's definition of LC [1].

Although research on LC is growing, we still know very little about the lived experiences of individuals with new‐onset pain following Covid‐19 infection. Understanding lived experience helps uncover how individuals make sense of and navigate their symptoms in everyday life—including the physical, emotional and social challenges that may not be captured through clinical assessments alone.

New‐onset pain is often persisting and widespread and can present in various forms—such as MSK pain, headache and neuropathic pain—frequently accompanied by reduced functional capacity and psychological impacts, including anxiety and depression [12]. Due to the diverse and complex nature of new‐onset LC pain and its significant impact on individuals' quality of life, a scoping review is appropriate for mapping the breadth and depth of available literature. While many qualitative studies have explored the broader experience of living with LC, we decided to concentrate on new‐onset pain since it is both prevalent and uniquely distressing. By focusing on this one area, we aimed to learn more about its impact on day‐to‐day living, which is often disregarded in more general studies.

While some symptoms of LC—such as ongoing pain and fatigue—share features with medically unexplained symptoms (MUS), which include fibromyalgia [18], chronic fatigue syndrome (CFS) and myalgic encephalomyelitis (ME), the relationship between LC and MUS appears unclear [15, 19]. Emerging research suggests symptom overlap between LC and conditions like ME/CFS, but the underlying pathophysiological mechanisms are not yet fully understood. As this review specifically examined the lived experiences of new‐onset LC pain and its impact on HRQoL, potential links between LC and MUS were not explored. Nonetheless, this remains an important area for future research.

This mixed‐methods scoping review will outline the main themes and gaps in the research, establish a foundation for future research, and offer recommendations for clinical and policy interventions to address new‐onset LC pain.

### 1.1 Objectives

1. To explore how individuals with any new‐onset pain following Covid‐19 describe their lived experiences, as reported in existing literature.
2. To examine how new‐onset pain following Covid‐19 impacts the HRQoL of those affected based on the current evidence.

### 1.2 Selection Criteria

Specific inclusion and exclusion criteria were created to select pertinent studies for a comprehensive and focused review. These were used to identify studies that investigate the lived experiences of new‐onset LC pain and its effects on HRQoL. Studies not meeting these criteria were omitted. The inclusion criteria are provided in Table 1.

**Table 1:** Inclusion criteria.

Inclusion criteria		Exclusion criteria	
1.	Studies that focus specifically on the lived experiences of new‐onset long Covid pain and its impact on the Health‐Related Quality of Life (HRQoL).	1.	Studies that do not specifically focus on new‐onset long Covid pain.
2.	Include patients with self‐reported new‐onset long Covid pain that begins at least 3 months after the initial Covid‐19 infection and persists for at least 2 months, in line with the WHO definition of the LC condition.	2.	Studies that include individuals who experience new‐onset pain within 3 months of the initial infection or where the diagnosis is not confirmed.
3.	Studies that report new‐onset pain co‐occurring with other LC symptoms.	3.	Studies that do not explore lived experiences or HRQoL in the context of new‐onset LC pain
4.	Studies published in English.	4.	Studies that focus solely on the prevalence of new‐onset LC pain without examining lived experiences or its impact on HRQoL
5.	Include primary research studies (quantitative, qualitative and mixed‐methods) and relevant unpublished articles such as preprints or theses.	5.	Studies published in languages other than English.
6.	Studies published between December 2019 (the emergence of Covid‐19) and June 2024.		

While the first objective focused on the lived experiences of new‐onset LC pain and the second on its impact on HRQoL, we included studies that addressed either or both of these aspects. Studies were first screened for relevance to new‐onset LC pain, then included if they explored lived experience or reported on HRQoL. We also included studies that focused solely on new‐onset pain and HRQoL, as these provide structured insights into how pain affects daily life, even if lived experiences were not explicitly discussed. This approach ensured alignment with both objectives while maintaining a broad yet focused inclusion strategy.

HRQoL was defined broadly to include both structured, quantitative assessments (e.g., EQ‐5D and SF‐36) and narrative or descriptive accounts of how new‐onset pain affected daily functioning, emotional well‐being and social life. This approach was designed to ensure alignment with both objectives and to reflect the multidimensional nature of HRQoL in the context of lived experience.

## 2 Methods

The scoping review was conducted and reported in adherence to the PRISMA‐ScR statement [20]. The methods were pre‐specified in a research protocol, which is published in the Open Science Framework (OSF) (OSF database registration number: OSF.IO/4PH8F: https://doi.org/10.17605/OSF.IO/4PH8F).

### 2.1 Search Strategy

The search strategy was developed in consultation with an information specialist. Search terms were determined with input from the research team. The following databases were searched: Web of Science, MEDLINE, Embase, CINAHL and PsycINFO. The search focused on studies involving human participants published in English between December 2019 and September 2023. The search began in December 2019 to ensure the review captured any early reports or emerging evidence related to new‐onset pain following Covid‐19 infection, including preliminary observations from the initial phase of the pandemic. An updated search was run in June 2024. A sensitive search was created in MEDLINE using database index terms and text words and adapted for other databases. The search strategy is available in Tables 2a and 2b.

**Table 2a:** Search terms.

Sl no.	Database	Concept 1 Lived experience	Concept 2 Pain	Concept 3 Long Covid	No. of records
1	MEDLINE Embase PsycINFO	“activities of daily living”/or “quality of life”/or exp Qualitative Research/or (Experience* or perception* or attitude* or “quality of life” or “well being” or “activities of daily living”). tw.	exp pain/or (((chronic or “long term” or persist* or prolong* or widespread or diffuse or recur* or refractory) adj3 pain) or pain or “chronic ache” or “post‐covid headache” or neuropath* or neuralgia or arthralgia or myalgia or radiculopathy). tw.	Post‐Acute Covid‐19 Syndrome/or (((long or chronic or persist* or residual or post or postacute or postinfec* or postvir* or relaps*) adj3 (covid* or coronavirus* or Cov or “SARS‐CoV‐2*” or “SARSCoV‐2*” or “SARSCoV2*” or “SARS‐CoV2*”)) or long‐haulers or postcovid*). tw.	MEDLINE: 423 Embase: 1068 PsycINFO: 33
2	CINAHL	(MH “Activities of Daily Living”) OR (MH “Quality of Life”) OR (MH “Qualitative Studies”) OR (Experience* OR perception* OR attitude* OR “quality of life” OR “well being” OR “activities of daily living”)	(MH “Pain+”) OR (((chronic OR “long term” OR persist* OR prolong* OR widespread OR diffuse OR recur* OR refractory) N3 pain) OR pain OR “chronic ache” OR “post‐covid headache” OR neuropath* OR neuralgia OR arthralgia OR myalgia OR radiculopathy)	(MH “Post‐Acute COVID‐19 Syndrome”) OR (((long OR chronic OR persist* OR residual OR post OR postacute OR postinfec* OR postvir* OR relaps*) N3 (covid* OR coronavirus* OR Cov OR “SARS‐CoV‐2*” OR “SARSCoV‐2*” OR “SARSCoV2*” OR “SARS‐CoV2*”)) OR long‐haulers OR postcovid*)	157
3	Web of Science	“activities of daily living” or “quality of life” or Qualitative Research or (Experience* or perception* or attitude* or “quality of life” or “well being” or “activities of daily living”)	pain or (((chronic or “long term” or persist* or prolong* or widespread or diffuse or recur* or refractory) Near/3 pain) or pain or “chronic ache” or “post‐covid headache” or neuropath* or neuralgia or arthralgia or myalgia or radiculopathy)	Post‐Acute Covid‐19 Syndrome or (((long or chronic or persist* or residual or post or postacute or postinfec* or postvir* or relaps*) Near/3 (covid* or coronavirus* or Cov or “SARS‐CoV‐2*” or “SARSCoV‐2*” or “SARSCoV2*” or “SARS‐CoV2*”)) or long‐haulers or postcovid*)	480
4		Concept 1 AND	Concept 2 AND	Concept 3	2161

*Note:* Ovid MEDLINE(R) and Epub Ahead of Print, In‐Process, In‐Data‐Review & Other Non‐Indexed Citations, Daily and Versions <1946 to September, 2023>.

*The index terms and text words remain consistent across MEDLINE, Embase and PsycINFO databases, presented in a single row and tailored for other databases.

**Table 2b:** Search terms.

Sl no.	Database	Concept 1 Lived experience	Concept 2 Pain	Concept 3 Long Covid	No. of records
1	MEDLINE Embase PsycINFO	“activities of daily living”/or “quality of life”/or exp Qualitative Research/or (Experience* or perception* or attitude* or “quality of life” or “well being” or “activities of daily living”). tw.	exp pain/or (((chronic or “long term” or persist* or prolong* or widespread or diffuse or recur* or refractory) adj3 pain) or pain or “chronic ache” or “post‐covid headache” or neuropath* or neuralgia or arthralgia or myalgia or radiculopathy). tw.	Post‐Acute Covid‐19 Syndrome/or (((long or chronic or persist* or residual or post or postacute or postinfec* or postvir* or relaps*) adj3 (covid* or coronavirus* or Cov or “SARS‐CoV‐2*” or “SARSCoV‐2*” or “SARSCoV2*” or “SARS‐CoV2*”)) or long‐haulers or postcovid*). tw.	MEDLINE:281 Embase: 573 PsycINFO: 23
2	CINAHL	(MH “Activities of Daily Living”) OR (MH “Quality of Life”) OR (MH “Qualitative Studies”) OR (Experience* OR perception* OR attitude* OR “quality of life” OR “well being” OR “activities of daily living”)	(MH “Pain+”) OR (((chronic OR “long term” OR persist* OR prolong* OR widespread OR diffuse OR recur* OR refractory) N3 pain) OR pain OR “chronic ache” OR “post‐covid headache” OR neuropath* OR neuralgia OR arthralgia OR myalgia OR radiculopathy)	(MH “Post‐Acute COVID‐19 Syndrome”) OR (((long OR chronic OR persist* OR residual OR post OR postacute OR postinfec* OR postvir* OR relaps*) N3 (covid* OR coronavirus* OR Cov OR “SARS‐CoV‐2*” OR “SARSCoV‐2*” OR “SARSCoV2*” OR “SARS‐CoV2*”)) OR long‐haulers OR postcovid*)	96
3	Web of Science	“activities of daily living” or “quality of life” or Qualitative Research or (Experience* or perception* or attitude* or “quality of life” or “well being” or “activities of daily living”)	pain or (((chronic or “long term” or persist* or prolong* or widespread or diffuse or recur* or refractory) Near/3 pain) or pain or “chronic ache” or “post‐covid headache” or neuropath* or neuralgia or arthralgia or myalgia or radiculopathy)	Post‐Acute COVID‐19 Syndrome or (((long or chronic or persist* or residual or post or postacute or postinfec* or postvir* or relaps*) Near/3 (covid* or coronavirus* or Cov or “SARS‐CoV‐2*” or “SARSCoV‐2*” or “SARSCoV2*” or “SARS‐CoV2*”)) or long‐haulers or postcovid*)	311
4		Concept 1 AND	Concept 2 AND	Concept 3	1284

*Note:* Ovid MEDLINE(R) and Epub Ahead of Print, In‐Process, In‐Data‐Review & Other Non‐Indexed Citations, Daily and Versions <1946 to June, 2024>.

* The search terms in the table were used for the initial and additional searches conducted in the database to identify relevant studies for the review.

Additional searches were conducted in consultation with the information specialist. The sources searched were: the WHO, Scottish Intercollegiate Guidelines Network (SIGN), Office for National Statistics (ONS), Centers for Disease Control and Prevention (CDC), National Institutes of Health (NIH), a health science preprint server (medRxiv.org), OSF, Google Scholar, Google and LitCOVID. Two studies were located using the search terms “long COVID,” “pain” and “quality of life” on Google Scholar and LitCOVID. These two studies were excluded because they indicated pain onset occurring within 12 weeks, which does not align with the WHO's definition of LC (symptom onset beyond 12 weeks). No relevant studies were found in other searched sources. No relevant conference reports, editorials or personal narratives on the experiences of new‐onset pain and its impact on the HRQoL were identified.

### 2.2 Source of Evidence Selection

All titles and abstracts were subjected to a dual screening process using the Covidence screening tool [21], with the primary scoping reviewer (M.P.) screening all articles. Other reviewers (A.G. and N.A.) also screened the same set by sharing the workload between them. Following this, M.P. screened all potentially eligible full‐text articles, while N.A. independently screened a random sample of three full‐text articles (representing a small proportion of the total full texts reviewed) for co‐validation. Disagreements at all stages were resolved through consensus.

### 2.3 Data Charting Process

M.P. extracted all evidence sources, while N.A. extracted a portion of the data to check for accuracy and completeness [22]. Based on the JBI guidance for scoping reviews, the data extraction tool was modified to capture information relevant to this study.

The tool included the details of the author and setting, aims and methods, demographic characteristics of participants, specifics of new‐onset LC pain and lived experiences, and the impact of pain on HRQoL. Characteristics of the eligible studies, including information about the study population and designs, were gathered. The data were extracted and categorised to generate a summary of outcomes and findings relevant to the review questions, such as the onset and duration of pain, lived experiences and HRQoL. Results were analysed and synthesised using a thematic approach [23]. Data were categorised into key themes: pain characteristics, pain experiences and the impact of pain on HRQoL. A descriptive and narrative synthesis was used to uncover patterns, differences and gaps in the studies and provide an overview of the new‐onset LC pain and its impact on HRQoL.

HRQoL was assessed using a range of validated tools across studies. These included generic instruments such as the EQ‐5D‐5L, SF‐12 and WHOQOL‐BREF, as well as the C19‐YRS scale, which captures symptoms, function and overall health status. Some studies used tools assessing mental health or pain‐related functioning (e.g., PHQ‐9, GAD‐7, BAI and FABQ), which represent components of HRQoL. While these tools varied in structure and scope, all captured domains relevant to overall health or functional impact. Therefore, synthesis focused on broad trends in HRQoL rather than direct comparison of scores.

In line with JBI guidance, no formal critical appraisal or risk of bias assessment was conducted, as scoping reviews are designed to map the breadth and nature of available evidence [24]. The primary goal was to overview or map the existing literature rather than synthesise findings based on methodological quality [20].

The modified tool and its components are available upon request, ensuring replicability for future research.

## 3 Results

### 3.1 Selection of Sources of Evidence

**Figure 1:**
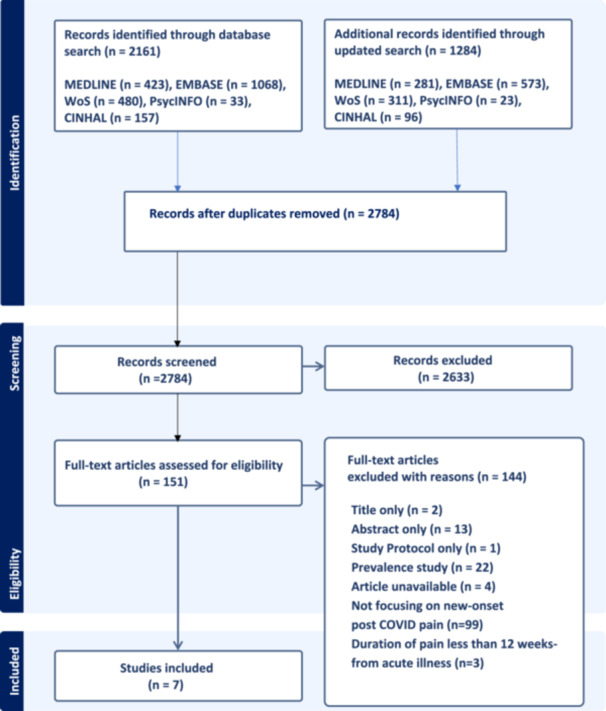
PRISMA Extension for Scoping Reviews (PRISMA‐ScR) diagram.

The initial literature search identified 2161 studies, and the updated search identified an additional 1284 studies. After duplicates were removed, a total of 2784 studies were screened for titles and abstracts, and 151 full‐text articles were assessed for eligibility. Finally, seven studies met the inclusion criteria and were included in the review. The PRISMA‐ScR diagram, explaining the study selection process, is illustrated in Figure 1.

Although the search strategy aimed to identify studies addressing both lived experience and HRQoL, no purely qualitative studies met the inclusion criteria. The included studies were predominantly quantitative, reflecting a gap in the literature rather than a limitation of the screening process.

### 3.2 Characteristics of Sources of Evidence

The seven included studies (*n* = 7) described self‐reported new‐onset LC pain and presented quantitative evidence [25, 26, 27, 28, 29, 30, 31]. Some studies administered structured or semi‐structured questionnaires through face‐to‐face or interviewer‐led formats, but these were used solely to collect quantitative data [27, 28, 30]. Of these, two studies used only questionnaires (*n* = 2) [25, 26], and one study used a questionnaire survey with clinical assessments [29].

Two of the seven studies were cross‐sectional case–control studies (*n* = 2) [25, 26], one was a case*–*control study (*n* = 1) [27], three were cross‐sectional studies (*n* = 3) [28, 29, 30] and one was an observational study (*n* = 1) [31]. The studies featured diverse sample sizes, ranging from 30 to 2507 participants, with 1523 females and 1782 males. All included studies focused on adult participants aged 18 years and over. No studies involving paediatric populations were identified during the search or screening stages, indicating a gap in the current evidence base regarding new‐onset LC pain in children and adolescents.

The geographic dispersion of the sample populations varied across different countries, including the United Kingdom [29], Spain [25, 26], France [28], Bangladesh [30] and Italy [31]. Nonetheless, the ethnicity of participants was not clearly specified in the studies, except in one [27]. Notably, the majority were conducted in Europe, with one in Asia and none in North America, which is further discussed as a limitation of this review.

Two studies (*n* = 2) included a healthy control group with no Covid‐19 infection [25, 26]. One study (*n* = 1) compared the assessment of pressure pain threshold and temporal summation of pain to age‐ and sex‐matched healthy subjects [31], Data collection was concluded for two studies (*n* = 2) in 2020 [27, 28], while one study (*n* = 1) completed data collection in 2021 [30]. Two studies (*n* = 2) completed data collection in 2022 [25], while two studies (*n* = 2) did not explicitly state the exact dates of data collection [29, 31].

### 3.3 Synthesis of Results

This review encompassed seven quantitative studies (*n* = 7), detailing the incidence, characteristics and effects of new‐onset pain symptoms on quality of life and daily activities. Nevertheless, none of the studies provided data on the personal experiences of individuals living with new‐onset pain. Although data on lived experiences were lacking, the studies offered significant insights into the incidence of new‐onset pain, its characteristics and its effects on HRQoL. These findings are provided in Tables 3a, 3b, 3c, 3d, 3e, 3f, 3g, followed by a summary of results.

**Table 3a:** Summary of characteristics of the studies and summary of the findings. *All participants exhibited similar socio‐demographic characteristics.

Study ID (country)	Aims and objectives, methods and methodology	Sample size, participant characteristics and date of data collection	Findings on new‐onset pain symptoms	Findings on Lived Experiences of new‐onset pain and its impact on HRQoL
Calvache‐Mateo et al. Pain and Clinical Presentation: A Cross‐Sectional Study of Patients with New‐Onset Chronic Pain in Long‐COVID‐19 Syndrome. Int J Environ Res Public Health. 2023a; 20(5):4049. doi:10.3390/ijerph20054049 Study setting: Spain	Aim: To assess new‐onset pain characteristics (intensity, interference, clinical presentation) in non‐hospitalised long Covid patients, comparing pain locations with successfully recovered Covid‐19 survivors and healthy controls. Study design: Cross‐sectional case–control study Methods: A questionnaire survey Assessment tools: Brief Pain Inventory, Short‐Form McGill Pain Questionnaire and Widespread Pain Index and EuroQol‐5 Dimensions 5 Levels, and Visual Analogue Scale.	Sample size: 213 participants LC group (71/53 F, 18 M) Successfully recovered Covid‐19 survivors (71/54 F, 17 M) Healthy controls (71/54 F, 17 M)	Incidence of pain: LC group: New‐onset pain—69.5% New‐onset headache—63.9%	Lived experiences: No specific data. HRQoL: LC Group: A higher score in pain and anxiety/depression. LC Group: A lower score in mobility, self‐care, usual activities and Visual Analogue Scale (*p* < 0.05).
			Pain characteristics (differences between the LC group, successfully recovered Covid‐19 survivors and healthy controls): Widespread pain (10.02 ± 5.93 vs. 1.16 ± 2.52 vs. 0.9 ± 1.68) Pain intensity (5.12 ± 2.28 vs. 0.93 ± 1.74 vs. 0.75 ± 1.48) Pain interference (5.78 ± 2.77 vs. 0.82 ± 1.87 vs. 0.51 ± 1.41) was observed.	
		Participants' age: 18 and above Date of data collection: May 2021 to April 2022 (3 months after the initial SARS‐CoV‐2 infection).		
			Pain sites: Neck (69.1%), legs (68%) and head (63.9%). Use of pain medication: LC group: Pain medication use (74.2%) and NSAIDs use (31.9%)	
			LC group: Women had 20% higher pain medication use Women used NSAIDs and metamizole (analgesic and antipyretic). Men used NSAIDs and paracetamol.	

**Table 3b:** Summary of characteristics of the studies and summary of the findings.

Study ID (country)	Aims and objectives, methods and methodology	Sample size, participant characteristics and date of data collection	Findings on new‐onset pain symptoms	Findings on Lived Experiences of new‐onset pain, and its Impact on HRQoL
Fernandez‐de‐las‐Penas et al. Myalgia as a symptom of hospital admission by severe acute respiratory syndrome coronavirus 2 infection is associated with persistent musculoskeletal pain as long‐term post‐Covid sequelae: a case‐control study. PAIN. 2021a; 162(12):2832‐2840. doi:10.1097/j.pain.0000000000002306 Study setting: Spain	Aim: Examine the association between Covid‐19‐related myalgia experienced at hospital admission and the presence of post‐Covid‐19 symptoms. Study design: Case–control study. Methods: Semi‐structured interviews with questionnaires Assessment tools: HADS and PSQI	Sample size: 738 (352 M/386 F) Participant characteristics: Hospitalised Covid‐19 survivors with (*n* = 369) and without (*n* = 369) myalgia at hospital admission. Age: Not specified. The average age is 61 years. Date of data collection: February 2020 to 31 March 2020 (7 months post‐hospital discharge).	Incidence of pain: Post‐Covid musculoskeletal (MSK) pain—284/738. New‐onset MSK pain—141/284 (73.2% prevalence). Pain sites: Spinal region	Lived experiences: No specific data.
				HRQoL: Anxiety, depressive level or sleep quality was not affected (data not shown). (The low levels of anxiety and depression in the sample could account for these findings)
	Hospitalisation and clinical data were collected from medical records.			

**Table 3c:** Summary of characteristics of the studies and summary of the findings.

Study ID (country)	Aims and objectives, methods and methodology	Sample size, participant characteristics and date of data collection	Findings on new‐onset pain symptoms	Findings on lived experiences of new‐onset pain and its impact on HRQoL
Martinez et al. Chronic pain characteristics in COVID‐19 survivors after an ICU stay: A cross‐sectional study. Anaesth Crit Care Pain Med. 2023; 42(1):101267. doi:10.1016/j.accpm.2023.101267 Study setting: France	Aim: To describe chronic pain, according to ICD‐11, among post‐hospital ICU Covid‐19 survivors. Study design: Cross‐Sectional Study Methods: Face‐to‐face interviews with questionnaires Assessment tools: A Brief Pain Inventory score was used to assess pain. Quality‐of‐life scores included physical and mental components of SF12.	Sample size: 143 (109 M, 34 F). Age: Not specified. Mean age—60 years Date of data collection: March 2020 to December 2020 (9 months post‐ICU discharge).	Incidence and type of pain:	Lived experiences: No specific data
			New‐onset chronic pain—54% (*n* = 77/143). Musculoskeletal pain (40%), post‐traumatic pain (34%), neuropathic pain (25%), visceral pain (13%) and orofacial pain (12.5%). 102 different forms of pain were reported.	HRQoL: General activities, work and sleep were affected. Low QoL scores with new‐onset pain (*z*‐score PCS12 −2.47 ± 1.14 vs. −1.57 ± 1.22, *p* < 0.0001; *z*‐score MCS12 −1.67 ± 0.83 vs. −0.92 ± 0.89, *p* < 0.0001). Higher scores for depression, anxiety and PTSD (38%) reported. 40% of new‐onset chronic pain cases required pain centre follow‐up, with one‐third referred to mental health specialists.
			Pain sites: Shoulders, chest, head and limbs. Factors linked to new‐onset chronic pain: Intubation.	

*ICD 11—International Classification of Diseases. *QoL—Quality of Life.

**Table 3d:** Summary of characteristics of the studies and summary of the findings.

Study ID (country)	Aims and objectives, methods and methodology	Sample size, participant characteristics and date of data collection	Findings on new‐onset pain symptoms	Findings on lived experiences of new‐onset pain and its impact on HRQoL
Calvache‐Mateo et al. Post‐Covid patients with new‐onset chronic pain 2 years after infection: Cross‐sectional study. Pain Management Nursing. 2023b; 24(5): 528‐534. doi.org/10.1016/j.pmn.2023.04.010 Study setting: Spain	Aim: To identify the clinical and psychosocial profile associated with pain in non‐hospitalised patients with post‐Covid‐19 syndrome Study design: Cross‐Sectional Case–Control Study. Methods: A questionnaire survey Assessment tools: Brief Pain Inventory (BPI), Central Sensitization Scale, Insomnia Severity Index (ISI), Tampa Scale for Kinesiophobia (TSK), Pain Catastrophizing Scale (PCS), Depression, Anxiety and Stress Scale (DASS), and Fear Avoidance Beliefs Questionnaire (FABQ).	Sample size: 170 participants Healthy control group (58, 69% F), Successfully recovered group (57, 66.7% F) Post‐Covid group (55, 70.9% F)	Pain characteristics: Higher levels of pain intensity in the post‐Covid group (5.07 ± 2.42), compared to the successfully recovered group (0.93 ± 1.74), and the healthy control group (0.66 ± 1.43). Higher levels of pain interference in the post‐Covid group (5.78 ± 3.14) compared to the successfully recovered group (0.82 ± 1.87) and the healthy control group (0.56 ± 1.63). Pain medication use: Post‐Covid group 76.4% Successfully recovered group 14% Healthy control group 10.3%. The most used drugs were NSAIDs, paracetamol and muscle relaxants. More patients attended physiotherapy than saw a psychologist.	Lived experiences: No specific reports.
		Age: Above 18 years Date of data collection: May 2021 to September 2022 (2 years post‐Covid‐19 infection).		HRQoL: Post‐Covid group had worse depression scores (*n* = 55: 25.38 ± 12.56) compared to the successfully recovered group (*n* = 58: 9.71 ± 11.66) and the healthy control group (*n* = 57: 9.47 ± 11.06). Post‐Covid group had worse fear‐avoidance beliefs (*n* = 55: 28.86 ± 13.76) compared to the healthy control group (*n* = 57: 7.98 ± 9.34) and successfully recovered group (*n* = 58: 10.43 ± 12.64) on the FABQ, including the physical activity subscale, work subscale and total score.

**Table 3e:** Summary of characteristics of the studies and summary of the findings.

Study ID (country)	Aims and objectives, methods and methodology	Sample size, participant characteristics and date of data collection	Findings on new‐onset pain symptoms	Findings on lived experiences of new‐onset pain and its impact on HRQoL
Khoja et al. Clinical characterisation of new‐onset chronic musculoskeletal (MSK) pain in Long COVID: a cross‐sectional study. J Pain Res. 2024; 17:2531‐2550. doi:10.2147/JPR.S466294. Study setting: United Kingdom	Aim: To investigate the clinical characteristics, underlying mechanisms and impact on function, psychological health and quality of life of individuals with new‐onset chronic MSK pain in patients with LC Study design: Cross‐Sectional Study Methods: A questionnaire survey Assessment tools: Brief Pain Inventory (BPI), Pain Self‐Efficacy Questionnaire (PSEQ), Pain Catastrophizing Scale (PCS), International Physical Activity Questionnaire—short form (IPAQ‐sf), Generalised Anxiety Disorder (GAD‐7), Patient Health Questionnaire (PHQ‐9) and EuroQol Five Dimensions health‐related quality of life (EQ‐5D‐5L). Clinical assessments: Timed Up and Go test (TUG), handgrip strength test, Covid‐19 Yorkshire Rehabilitation Scale (C19‐YRS).	Sample size: 30 (11 M, 19 F) Participants with LC and new‐onset chronic MSK pain	Incidence of new‐onset pain: All (100%) participants had new‐onset chronic MSK pain. Pain frequency and quality: 90% experienced continuous pain, varying in intensity, mostly described as dull aching, with some reporting sharp stabbing pain. Pain intensity: The mean BPI total pain severity score was 5.3 ( ± 1.4), with 54.2% in the moderate pain category. Pain interference: The mean pain interference score was 6.2 ( ± 2.2), with 84.0% reporting high interference. Among the pain interference items, the most impacted areas were enjoyment of life (7.3 ± 2.4), normal work (6.5 ± 2.5) and mood (6.4 ± 2.8).	Lived experiences: No specific data. HRQoL: Anxiety: Pre‐Covid: 60% (1.7 ± 1.9), Post‐Covid: 92% (5.7 ± 2.9). Depression: Pre‐Covid: 32% (1.2 ± 2.2), Post‐Covid: 68% (4.0 ± 3.3). PTSD: Pre‐Covid: 20% (0.4 ± 1.0), Post‐Covid: 60% (3.2 ± 3.3). Mobility: Pre‐Covid: 20% (0.4 ± 1.0), Post‐Covid: 76% (4.6 ± 3.4). Personal care: Pre‐Covid: 8% (0.3 ± 1.2), Post‐Covid: 52% (2.1 ± 2.7). Other ADLs: Pre‐Covid: 20% (0.3 ± 0.7), Post‐Covid: 92% (6.6 ± 2.6). Functional disability score (0–50): Pre‐Covid: 1.5 ± 3.0, Post‐Covid: 22.8 ± 11.3
		Date of data collection: Not specified (The mean duration from the onset of MSK pain to the study evaluation was 519.1 days ( ± 231.7, which is more than 3 months).		
			Pain sites: Knees (70%), shoulders (63%), cervical spine (60%) and lumbosacral region (57%). Those without widespread pain (10%) experienced pain in at least four body areas	Physical activity levels: 64% (*n* = 16) of the participants were categorised as ‘inactive’. 28% (*n* = 7) were classified as ‘minimally active’. Only 8% (*n* = 2) of participants reached the criteria of ‘HEPA active’ (Health‐Enhancing Physical Activity)

**Table 3f:** Summary of characteristics of the studies and summary of the findings.

Study ID (country)	Aims and objectives, methods and methodology	Sample size, participant characteristics and date of data collection	Findings on new‐onset pain symptoms	Findings on lived experiences of new‐onset pain and its impact on HRQoL
Kabir et al. Clinical presentation of post‐COVID pain and its impact on quality of life in long COVID patients: a cross‐sectional household survey of SARS‐CoV‐2 cases in Bangladesh. BMC Infectious Diseases 2024; 24(1):1‐13. DOI: 10.1186/s12879‐024‐09267‐3 Study setting: Bangladesh	Aim: To elicit the clinical presentation of pain and determine the relationships between QoL and pain in LCS. Study design: Cross‐Sectional Study. Methods: Face‐to‐face interviews with semi‐structured questionnaires Assessment tools: Covid‐19 Yorkshire Rehabilitation Scale and WHOQOL‐BREF.	Sample size: 2507 22.45% (563/2507) of participants with LC symptoms, while 77.54% (1944/2507) were asymptomatic.	Incidence of pain: Between 0.03% and 3.1% Types of pain: Chest pain 2.4% (95% CI, 1.8%–3.1%), joint pain 2.8% (95% CI, 2.2%–2.35%), muscle pain 3.1% (95% CI, 2.4%–3.8%), headache 3.1% (95% CI, 2.4%–3.8%) and abdominal pain 0.3% (95% CI, 0.01%–0.5%). Pain intensity: The severity of the pain in the Brief Pain Inventory was 8.22 ± 4.09 for severe pain and 9.03 ± 7.661 for pain interference. Predictors of different pain: Abdominal pain—more LC symptoms. Headache—vaccination with a booster dose. Muscle pain/Joint pain: Longer duration of LC symptoms, more LC symptoms and poor quality of life. Chest pain: Longer duration of LC, severe LC symptoms, poor psychological health. Joint pain/muscle pain: Longer duration of LC, more LC symptoms, poor quality of life. Headache: Vaccination with a booster dose, more LC symptoms, poor physical health.	Lived experiences: No specific data.
		Age: 18 years and above. Date of data collection: July 2021 to December 2021		HRQoL: Higher pain scores are associated with lower QoL in all measured domains. Physical health and pain scores: −0.783 (*p* < 0.01) Psychological health and pain scores: −0.804 (*p* < 0.01) Social relationships and pain scores: −0.802 (*p* < 0.01) Environmental health and pain scores: *p* < 0.01

*There is a discrepancy in the reported prevalence of pain in LC: the abstract states 0.1%–3.1%, while the discussion reports 0.3%–3.1%. Headaches are noted as 3.1% in ‘Abstract’ and ‘Results’, but 0.3% in the ‘Discussion’. The headache prevalence should be 0.3% as it appears in two sections. The study uses the WHO definition of LC: developing or continuing new symptoms after 12 weeks of Covid‐19 and persisting for at least 2 months without another diagnosis.

**Table 3g:** Summary of characteristics of the studies and summary of the findings. *Late‐onset pain is being interpreted as new‐onset based on the cohort's presentation of symptoms.

Study ID (country)	Aims and objectives, methods and methodology	Sample size, participant characteristics and date of data collection	Findings of new‐onset pain symptoms	Findings of lived experiences of new‐onset pain and its impact on HRQoL
Baroni et al. Fatigue can influence the development of late‐onset pain in post‐COVID‐19 syndrome: an observational study. European journal of pain 2024;28(6):90 1‐912 2023. doi: 10.1002/eip.2228 Eur J Pain. 2024;28:901‐912 Study setting: Italy	Aim: To assess the incidence of 1‐year post‐Covid‐19 pain to identify potential contributing factors. Study design: Observational Study. Methods: Quantitative questionnaire survey Assessment tools: Covid‐19 Yorkshire Rehabilitation Scale (C19‐YRS) Numeric Pain Rating Scale (NPRS), Leeds Assessment of Neuropathic Symptoms and Signs (LANSS), the Central Sensitisation Inventory (CSI). the Pain Catastrophizing Scale (PCS), the Tampa Scale of Kinesiophobia (TSK), the Pressure Pain Threshold (PPT) and Temporal Summation (TS). the Beck Anxiety Inventory (BAI), the Patient Health Questionnaire‐9 (PHQ‐9), and the Impact Event Scale‐Revised (IESR).	Sample size: 67 (45 M and 22 F) hospitalised patients with Covid‐19	Incidence of pain: Almost one out of three patients hospitalised for Covid‐19 developed pain 1 year later 20/67 subjects presented increased pain intensity > 2 points at the 52‐week C19‐YRS pain assessment compared to the pre‐Covid condition and the 12‐ and 26‐week evaluations. 14/20 patients who showed an increase in pain intensity at 52 weeks (showed maintenance or reduction of pain intensity between 12 and 26 weeks). 3/14 patients developed new‐onset pain at 52 weeks (with no pain before infection). Pain sites: Of the 18/20 participants with pain at 52 weeks, most had pain in the shoulder, knee and lumbar spine Risk factors: Fatigue Pain characteristics: Most of the patients did not show nociplastic or neuropathic mechanisms of pain.	Lived experiences: No specific data HRQoL: Worse outcomes in fatigue, anxiety, mobility, daily activities and overall health perception.
		Age: 18 and over. Mean age—66.4 ± 10.6 years No one was vaccinated against Covid‐19 Date of data collection: Not specified		

### 3.4 New‐Onset Pain Symptoms

The incidence of new‐onset pain in people experiencing LC varied among selected studies, while one study did not report the incidence of new‐onset pain [26]. New‐onset pain was reported by 69.5% of patients with LC [25] and was mostly MSK (73.2%) [27], affecting common sites such as the head, neck, shoulders, spine, knees, lower back, chest and legs [25, 27, 28, 29, 30]. While one study [27] reported 73.2% (*n* = 141/284) of the incidence of new‐onset MSK pain, another study found that all their participants (100%, *n* = 30) experienced new‐onset MSK pain [29]. This variation in pain locations suggests the possibility of multiple pain types coexisting within individuals.

Certain studies, such as Fernandez‐de‐las‐Penas et al. [27], focused on hospitalised patients, primarily Caucasian, which restricts the applicability of the findings to non‐hospitalised or ethnically varied populations. The intensity and interference of pain varied widely [25, 29, 30]. Women with new‐onset pain reported 20% higher medication use, particularly nonsteroidal anti‐inflammatory drugs (NSAIDs) and metamizole, to manage symptoms [25]. However, this study used assessment scales such as the Widespread Pain Index (WPI) and EuroQol‐5 Dimensions (EQ‐5D‐5L), which, though validated in other populations, have not been specifically validated for LC patients, limiting the robustness of the results [25].

Khoja et al. [29] reported that all participants (100%) in the group had new‐onset MSK pain, which was persistent and greatly interfered with the activities of daily living. Small sample sizes in some studies [25, 29, 31] limited the generalisation of these findings to larger populations. While some studies met the WHO timing criterion for LC (onset of pain 3 months after infection), they did not confirm whether pain persisted for the required minimum duration of 2 months [27, 31].

One study found an association between the presence of muscle pain during the acute phase of Covid‐19 and the later development of LC MSK pain [27]. This may serve as an early clinical indicator for identifying individuals at risk of developing new‐onset LC pain.

Baroni et al. [31] reported late‐onset pain developing 1 year post‐infection. Based on the cohort's presentation of symptoms, these cases were classified as new‐onset pain, but attempts to obtain confirmation from the contact authors were unsuccessful.

New‐onset pain was consistently linked to lower HRQoL across all studies [25, 26, 27, 28, 29, 30, 31]. Patients with new‐onset LC pain reported notable declines in both physical and mental well‐being, with heightened levels of anxiety, depression [25, 26, 27, 28, 29, 30, 31] and PTSD [28, 29]. For example, patients admitted to the intensive care unit (ICU) during their acute phase of Covid‐19 developed new‐onset LC pain and exhibited significantly lower HRQoL scores and higher levels of psychological distress [28]. Likewise, there was a strong correlation between higher pain scores and diminished quality of life across physical, psychological and social aspects [30]. Nonetheless, not every study employed the same scales for measuring HRQoL.

### 3.5 Psychosocial and Clinical Factors

This review highlighted that fatigue is a major risk factor for developing new‐onset pain [31]. However, the study fails to establish a causal relationship between fatigue and the onset of pain. Additionally, Calvache‐Mateo et al. [26] found that people in the post‐Covid group showed elevated levels of depression and fear‐avoidance beliefs compared to those who had fully recovered or were in good health, highlighting the significant psychological burden associated with new‐onset pain in LC patients. Nonetheless, the small sample sizes again restrict the ability to make broad conclusions.

The findings of this review illustrate that new‐onset pain is a prevalent and significant symptom of LC, greatly impacting both physical and mental health. Despite differences in study designs and limitations in their generalisability, a consistent finding across all studies was the profound negative impact on the quality of life of LC patients.

## 4 Discussion

### 4.1 Summary of Key Findings

This scoping review reports significant effects of new‐onset pain following Covid‐19 on physical, psychological and social well‐being. Although qualitative data on lived experiences are limited, quantitative studies provide a significant understanding of the incidence of pain, its characteristics, use of pain medication, effects of pain on daily activities, and psychological well‐being. The review identifies gaps in the existing research, highlighting the need for further studies to understand the personal experiences of those suffering from new‐onset LC pain, emphasising the urgent need for targeted interventions and support to enhance their HRQoL. Details are summarised in Tables 3a, 3b, 3c, 3d, 3e, 3f, 3g.

### 4.2 New‐Onset Pain Symptoms and Medications

The findings align with previous research on post‐viral infections, which also report MSK pain as a common outcome, with similar patterns observed among survivors of Ebola virus disease [32]. Common areas of pain identified in this review include the head, shoulders, chest, spinal region, abdomen and knees [25, 28, 29, 30, 31], which corresponds with other research highlighting some of these areas as typical for LC pain [33]. This is also consistent with other research highlighting the joints and lower back as common pain sites in LC patients [34, 35]. However, Mills et al. [36] indicated that MSK pain is most commonly found in the lower limbs and lumbar region, implying that different types of new‐onset pain might emerge within the same patient experiencing LC syndrome. Variability in pain sites and characteristics across different studies could be attributed to differences in participant demographics, study designs and pain assessment methods, highlighting the need for more nuanced research.

This review found that the development of MSK post‐Covid pain is associated with experiencing muscle pain as a symptom during the onset of Covid‐19 infection and hospitalisation [27]. Early identification of muscle pain may help clinicians recognise individuals at risk of developing chronic LC MSK pain [27]. MSK pain is important to consider because it is not commonly regarded as one of the most bothersome symptoms of Covid‐19 compared to other related symptoms such as dyspnoea, fever or chest pain. Although pain intensity varied across the studies reviewed [26], the emergence of new‐onset MSK pain in previously healthy individuals could become a risk factor for developing chronic pain if not properly addressed.

The review reported significant use of pain medications, especially NSAIDs and analgesics, among LC patients [26]. Women reported 20% higher drug intake [26], which aligns with known gender disparities in pain experiences. Studies report that women are more likely than men to report greater pain severity [37, 38] and use more pain medication [39, 40]. These differences may be affected by biological factors, such as hormonal variations, and social factors, with increased societal acceptance of women's expression of pain and higher rates of chronic pain diagnoses among women [41]. Women frequently face the challenge of coping with pain while managing multiple demands [42], such as familial duties, professional commitments and household responsibilities [41]. This burden can greatly hinder pain recovery, affecting overall well‐being [41].

### 4.3 Impact on HRQoL

The review showed that people living with LC symptoms tend to have a reduced quality of life than those who have completely recovered from Covid‐19 and the general population. Similarly, Huynh et al. [43] found that people recovering from Covid‐19 reported a lower quality of life compared to the general population. The review indicated higher levels of pain, anxiety and depression, along with decreased mobility, all of which are strongly linked to diminished quality of life across physical, psychological, social and environmental aspects [25, 26, 28, 29, 30, 31]. These results underscore the profound effects of pain on different aspects of well‐being.

The prevalence of PTSD was significant, especially among hospital ICU survivors, with 38% reporting symptoms that negatively affected their quality of life [24]. Numerous other studies indicate that LC is associated with a reduced quality of life [44, 45, 46, 47], with PTSD recognised as a possible underlying factor [48]. The trauma experienced by some patients during hospitalisation may arise from the severity of the illness or circumstances such as witnessing a family member's death from Covid‐19, both of which can potentially induce PTSD [49]. Research indicates that insufficient social support after recovery from Covid‐19 correlates with higher levels of PTSD [50]_._ It is noteworthy that the development of PTSD may not be affected by variables such as age, sex, length of hospitalisation or time since discharge, as indicated by Chang and Park [51]. This review revealed contrasting findings, indicating no significant association between new‐onset pain and anxiety, depression or sleep quality [27]. This discrepancy may result from the differences in study populations or the assessment methods used.

According to the results of this scoping review, most people who experience new‐onset pain after recovering from Covid‐19 have a reduced quality of life, which poses significant challenges for them and their healthcare providers.

### 4.4 Implications for Practice and Future Research

This review highlights several implications for both clinical practice and future research. Clinically, there is a need to recognise MSK pain as a potentially debilitating and chronic symptom of LC. The risk of long‐term consequences may be reduced by early detection and focused treatment of MSK pain, especially in patients who experienced muscular pain during an acute illness. Healthcare professionals need to be able to assess and manage new‐onset pain with a holistic approach, not just the physical symptoms, but also the psychological and social impacts of pain. Differences in how men and women report pain and use medication highlight the importance of tailoring care to the individual, recognising biological factors, social roles and the demands of daily life, including caregiving responsibilities.

From a research point of view, there's a clear need to understand how different genders experience and manage new‐onset LC pain. This includes looking at how pain changes over time, how people respond to treatments, and the methods they use to cope. Investigating how pain affects everyday life, including employment, family responsibilities and recovery, is also important, as it can help create interventions that consider different genders. To ensure that studies reflect what matters most to those affected, future research should meaningfully involve people with lived experience in shaping research questions and interpreting findings.

### 4.5 Strengths of the Review

This review offers a comprehensive overview of the incidence, characteristics and impacts of new‐onset pain following Covid‐19, highlighting its significant effects on HRQoL. Despite some limitations, it illustrates the major negative effects of new‐onset LC pain on physical, mental and social well‐being. By incorporating data from diverse populations and study designs, the review highlights key gaps, such as the need for qualitative, mixed‐methods or longitudinal research and for testing the accuracy of assessment tools in LC populations. It also serves as a reminder of the need for appropriate support for people with new‐onset LC pain and points to areas for future research.

### 4.6 Limitations of the Review

This scoping review is constrained by the lack of qualitative evidence on the lived experiences of individuals with new‐onset LC pain, hence limiting the understanding of its effects on daily life and mental health. The lack of longitudinal studies limits the understanding of pain progression and HRQoL over time. Small sample sizes in some studies lower the strength of the findings [29, 31], and the studies reviewed lacked diversity, with one study focusing mainly on Caucasian participants [25], which makes it harder to apply the results to different groups. The geographic scope was also limited, with no data from North America, a region with a large and varied population that was significantly impacted by Covid‐19. This makes it more difficult to understand the global picture and to relate the findings to different healthcare systems and cultural settings. Studies that focused only on hospitalised [27, 28, 31] or only on non‐hospitalised patients [25, 26] also make it harder to understand the full range of pain experiences, especially across varying levels of severity in new‐onset LC pain.

An additional limitation of the review is the lack of patient and public involvement (PPI). Although scoping reviews usually aim to bring together the existing literature, PPI could have contributed to shaping the direction and relevance of the review, especially by offering insights into lived experiences that may not be captured in published studies. Their involvement may also have improved the presentation of findings, making them more accessible and meaningful. This is a learning point that will be carried into the next phases of the research, where PPI will play a more active and collaborative role.

Focusing on HRQoL as a separate objective made it possible to include studies that examined the impact of new‐onset pain using structured tools, even when lived experiences were not explored in depth. This approach helped show both measurable health impacts and subjective experiences, though it may have led to the exclusion of studies that described pain‐related experiences more narratively without clearly referencing HRQoL.

No studies on the review topic were identified in paediatric populations, highlighting a gap in the evidence on new‐onset LC pain in children and adolescents.

## 5 Conclusion

The findings of this scoping review highlight that new‐onset pain is a common symptom following Covid‐19 and has a major impact on the HRQoL. New‐onset pain affects the physical, psychological and social well‐being of those with LC. Nonetheless, the lived experiences of individuals with new‐onset LC pain remain underexplored, leaving a gap in the existing literature. Research is needed to explore these gaps using mixed‐methods or qualitative approaches to better understand the impacts of new‐onset pain.

Research priorities identified by patients, patient charities and carers include understanding the trajectory of LC, addressing muscle‐related symptoms and supporting mental well‐being [52]. Therefore, future research needs to incorporate these priorities and be conducted in partnership with patients and the public through co‐designed projects for the development of effective, patient‐centred and multidisciplinary interventions that enhance the HRQoL of those living with new‐onset LC pain.

## Author Contributions

**Minimol Paulose:** conceptualisation, data curation (collated data in Tables in 3a–3g), writing – original draft, writing – review and editing. **Aileen Grant**, **Nicholas Norman Adams**, and **Kathryn R. Martin:** writing – review and editing.

## Conflicts of Interest

The authors declare no conflicts of interest.

## Data Availability

Data are available from the corresponding author upon reasonable request.
